# Chronic cough associated with Crohn's disease

**DOI:** 10.1186/1745-9974-6-6

**Published:** 2010-08-08

**Authors:** Shoaib Faruqi, Ged Avery, Alyn H Morice

**Affiliations:** 1 Department of Respiratory Medicine, Castle Hill Hospital, Castle Road, Cottingham, HU16 5JQ, UK; 2 Department of Radiology, Castle Hill Hospital, Castle Road, Cottingham, HU16 5JQ, UK

## Abstract

A 62-year-old man presented with chronic dry cough. He was known to have Crohn's disease which was in remission. A plain chest radiograph demonstrated bilateral apical infiltrates. A HRCT of the chest showed normal proximal airways. Stenosis of medium size airways was present with post-stenotic dilation. These dilated peripheral bronchi appeared fluid filled. Patchy areas of consolidation were seen as well. These changes were thought to be due to Crohn's disease involving the lungs and responded well to treatment with cortico-steroids. We report this uncommon radiological association with Crohn's disease.

## Background

Clinically relevant respiratory manifestations of inflammatory bowel disease are very uncommon. They are reported more commonly in association with Ulcerative Colitis and less often with Crohn's disease. The most frequent respiratory manifestation of inflammatory bowel disease is bronchiectasis. We report a case of chronic dry cough in association with Crohn's disease with interesting associated radiology and good response to treatment with steroids.

## Case report

**Figure 1:**
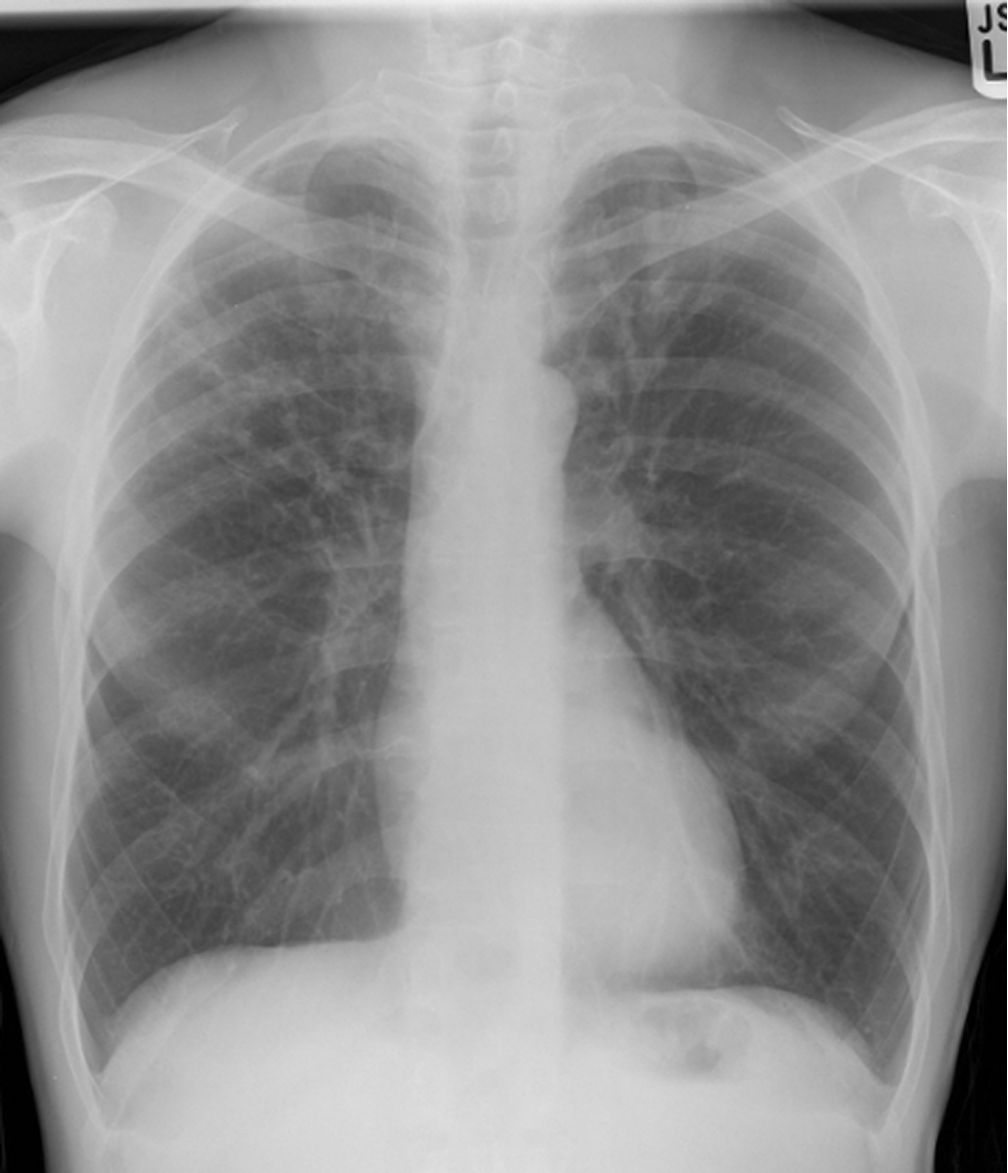
**A plain chest radiograph demonstrating infiltrates in both the apices**.

A 62-year-old man presented with dry cough of five years duration with no associated breathlessness or wheezing. He did not report a post nasal drip or systemic symptoms. He was diagnosed to have Crohn's disease with gastro duodenal involvement ten years earlier. The diagnosis was established based upon typical features on a duodenal biopsy. He was treated with prednisolone and mesalazine. The Crohn's disease was in remission in less than a year following which prednisolone and mesalazine were discontinued. He was continued on treatment with a proton pump inhibitor. He worked as a university lecturer. He was an ex-smoker of ten pack years and had stopped smoking ten years earlier. On examination he did not have cyanosis, digital clubbing or significant lymphadenopathy. Examination of the respiratory system was unremarkable. A chest radiograph demonstrated bilateral apical infiltrates (Figure 1). A full blood count, biochemical profile, angiotensin converting enzyme levels and total as well as specific immunoglobulins were all normal.

**Figure 2:**
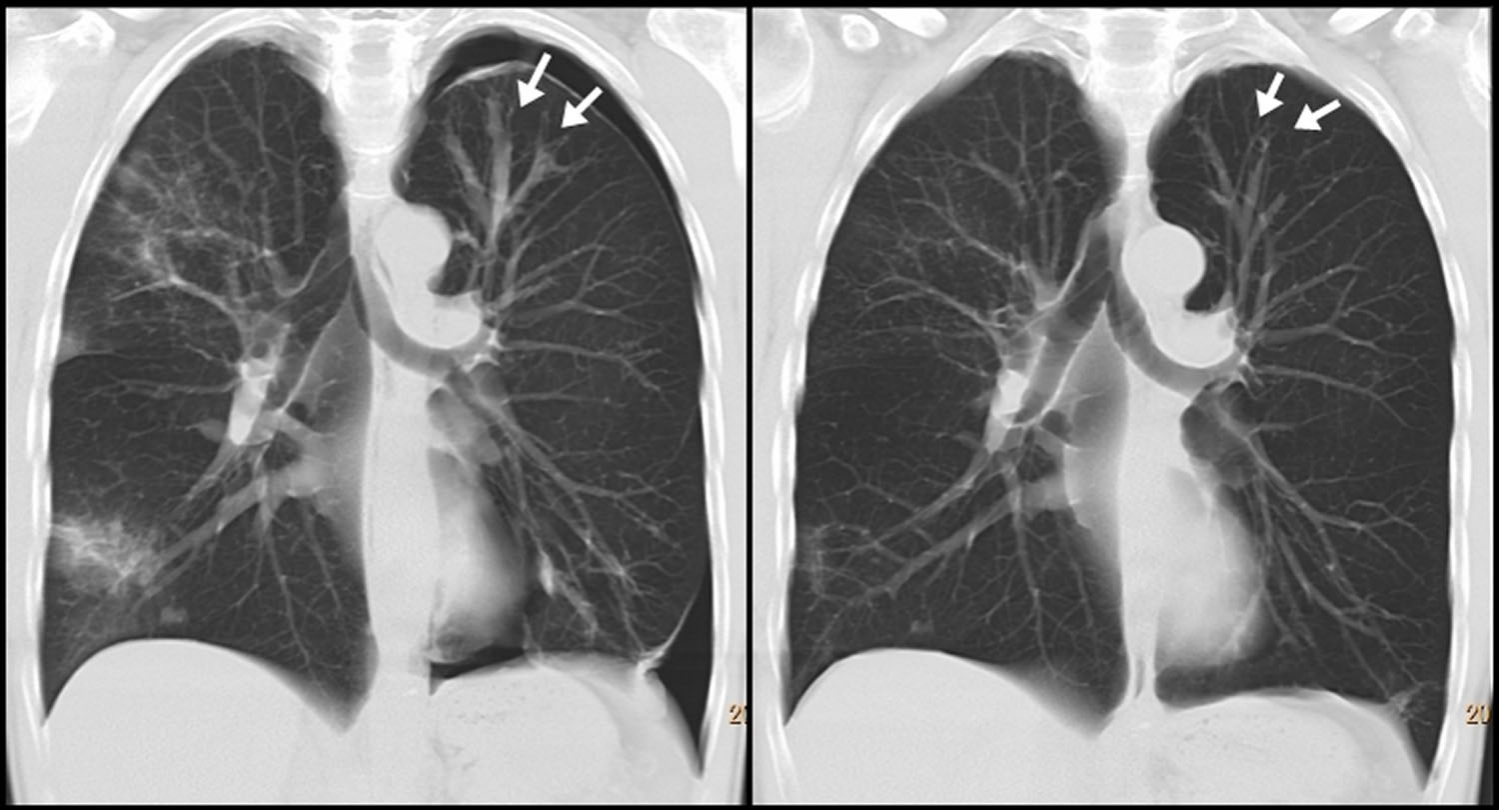
**A reformatted coronal CT image demonstrating dilated fluid filled bronchi in the panel on left**. The bronchi can be followed centrally. They narrow down and then appear normal. This is well seen in the left upper lobe (arrows). Areas of patchy consolidation are seen bilaterally. A small left pneumothorax is seen which was a complication of the trans-bronchial biopsy. Panel on the right shows a reformatted coronal CT image at the same level six weeks later. The dilated bronchi seen on the earlier scan have markedly improved. This is clearly demonstrated in the left upper lobe (arrows). The areas of consolidation have improved as well. The pneumothorax has now resolved without need for drainage.

**Figure 3:**
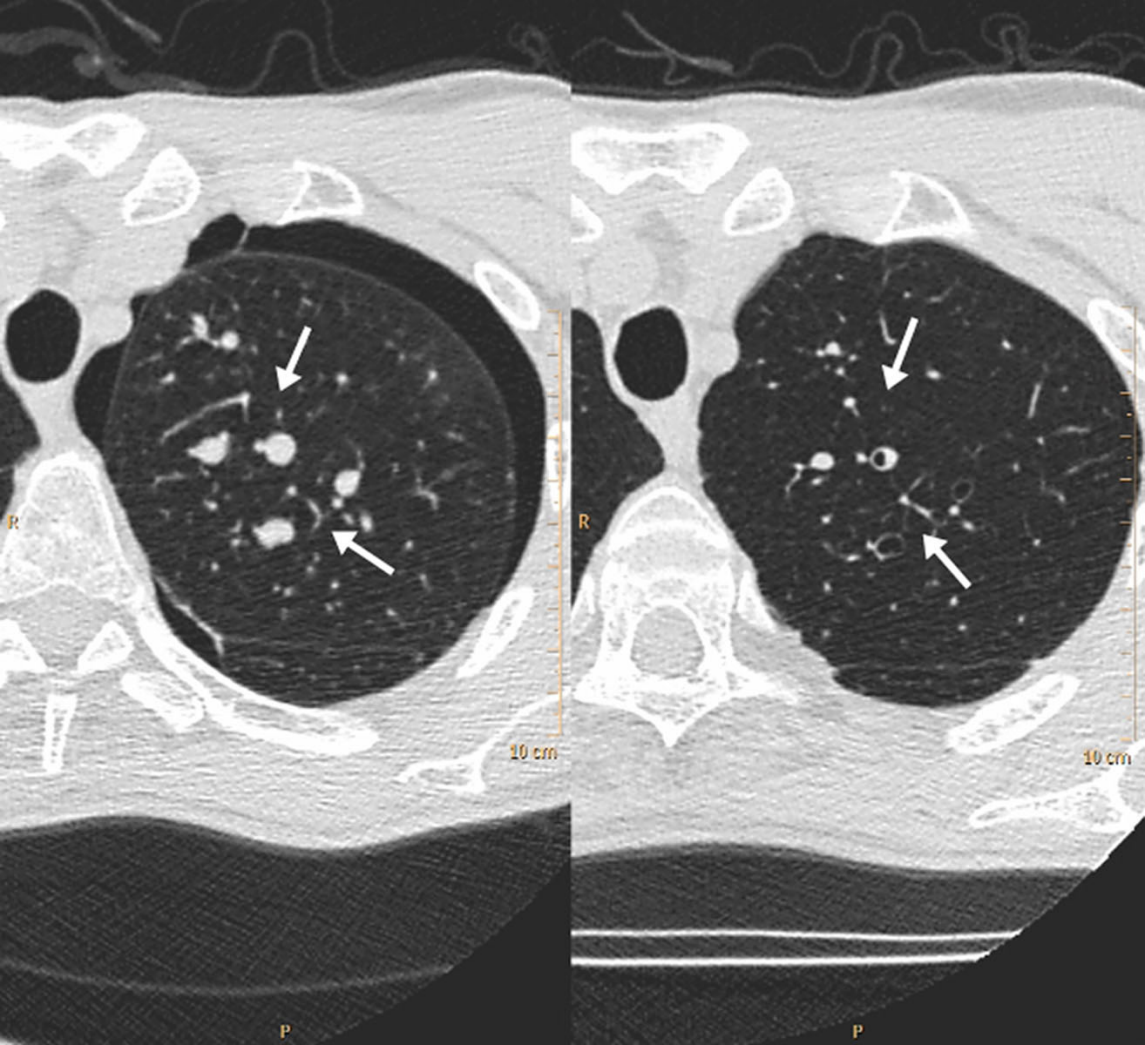
**CT image at the same level before and following treatment at six weeks**. Arrows annotate the fluid filled dilated bronchi which demonstrate improvement following treatment.

A fibre optic bronchoscopic examination was macroscopically normal. The appearance of the trachea and the bronchial tree was entirely normal. Based on the chest radiograph, a bronchial wash as well as bronchial and trans-bronchial biopsies and were performed from the left upper lobe. The bronchial wash was sterile and negative for acid fast bacilli on stain and culture. The bronchial biopsy showed evidence of a mild inflammatory cell infiltrate, including eosinophils, in the sub epithelial connective tissue. The trans-bronchial lung biopsy was normal. The trans-bronchial biopsy was complicated by a small pneumothorax which did not need any intervention. A high resolution computed tomography (CT) scan showed a normal trachea and normal proximal airways which narrowed and then dilated peripherally. These dilated peripheral bronchi appeared fluid filled. These changes were seen bilaterally, well demonstrated in the left upper lobe (Figure 2). Areas of patchy air space shadowing were seen bilaterally. Adjacent to these areas of consolidation small branching opacities consistent with small airways involvement were also noted. It was thought that these changes were due to Crohn's disease and treatment with prednisolone was initiated at a dose of 10 mg once daily. He responded well to treatment with complete resolution in symptoms. A CT scan done 6 weeks following initiation of treatment showed good improvement in the changes seen earlier (Figure 2, 3). Nine months later prednisolone was tapered and stopped. However he relapsed on discontinuing prednisolone and this had to be re-instituted. His symptoms resolved with the re-introduction of prednisolone. He remains asymptomatic on treatment with 2.5 mg of prednisolone along with inhaled budesonide.

## Discussion and conclusion

The Inflammatory Bowel Diseases (IBD), Ulcerative Colitis (UC) and Crohn's disease (CD), are known to have multiple extra intestinal manifestations with as many as 36% of cases having at least one [1]. Although association between respiratory disease and IBD has been observed more than three decades ago, clinically significant respiratory manifestations of IBD are uncommon [2,3]. Any part of the lung and its vasculature may be involved in association with IBD. Large airways disease is the commonest site of lung involvement in IBD. In a recent review these accounted for 39% of the cases of which two thirds comprise bronchiectasis. Bronchiectasis is most commonly observed in UC, predominates in women and more common in non-smokers. Interestingly flare up of bronchiectasis has been observed within a year following colectomy [4,5]. This transfer of the inflammatory process from the gastro intestinal tract to the lungs has been suggested as evidence for causal link between the two [6]. The common origin of the lung and the gastro intestinal tract from the primitive foregut and similarities in tissue structure suggest a patho-physiologic reason for lung involvement in IBD.

Clinically smaller airways disease in IBD is rare and involvement is both at a younger age and at an early point in the disease course. Pathologically, bronchiolitis is most commonly reported. Bronchiolitis obliterans organizing pneumonia (BOOP) is the most common parenchymal lung manifestation reported in association with IBD. In the majority of cases the association is with UC. As with idiopathic BOOP, it responds well to corticosteroid therapy. Several other parenchymal lung diseases such as other interstitial pneumonias and eosinophilic pneumonias as well as pulmonary nodules have been reported. Pulmonary nodules are rare and can be necrobiotic or granulomatous [4,5,7,8].

Although the lung manifestations of IBD have been well described in literature, our patient was unique in the indolent presentation as well as the distinctive radiological features. Large airways involvement in the form of severe tracheo-bronchial stenosis with marked inflammation has been observed in CD [8]. In our patient stenosis was seen in medium size airways. Bronchial biopsy showed evidence of inflammation which is the most prevalent involvement in IBD [5]. The location of stenosis in the medium size bronchi lead to the unique radiological picture of dilated, fluid filled peripheral airways seen on the CT scan. Patchy areas of consolidation seen could represent BOOP. In the context of IBD, associated lung diseases respond well to corticosteroid treatment. The dosage, duration and route of administration are empirical and based on clinical experiences. As the symptoms of our patient were mild in nature we started treatment with a relatively low dose of prednisolone to which he responded very well. However stopping prednisolone resulted in a relapse of his symptoms necessitating a small maintenance dose along with inhaled budesonide. He remains well on the above treatment.

## Competing interests

The authors declare that they have no competing interests.

## Authors' contributions

AHM and SF were the clinicians and GA the radiologist managing the case. SF drafted the initial manuscript. All authors have read and approved the final manuscript.

## Consent

Written informed consent was obtained from the patient for publication of this case report and accompanying images. A copy of the written consent is available for review by the Editor-in-Chief of this journal.
